# Sports Heart Monitors as Reliable Diagnostic Tools for Training Control and Detecting Arrhythmias in Professional and Leisure-Time Endurance Athletes: An Expert Consensus Statement

**DOI:** 10.1007/s40279-023-01948-4

**Published:** 2023-10-31

**Authors:** Robert Gajda, Jacek Gajda, Miłosz Czuba, Beat Knechtle, Wojciech Drygas

**Affiliations:** 1 Center for Sports Cardiology at the Gajda-Med Medical Center in Pułtusk, 06-100 Pułtusk, Poland; 2 Department of Kinesiology and Health Prevention, Jan Dlugosz University, Czestochowa, Poland; 3 Faculty of Rehabilitation, Józef Piłsudski University of Physical Education in Warsaw, Warsaw, Poland; 4 Institute of Primary Care, University of Zurich, Zurich, Switzerland; 5 Medbase St. Gallen am Vadianplatz, St. Gallen, Switzerland; 6 Department of Epidemiology, Cardiovascular Disease Prevention, and Health Promotion, The Cardinal Stefan Wyszynski National Institute of Cardiology, Warsaw, Poland; 7 Lazarski University, Warsaw, Poland

## Abstract

There are countless types of portable heart rate monitoring medical devices used variously by leisure-time exercisers, professional athletes, and chronically ill patients. Almost all the currently used heart rate monitors are capable of detecting arrhythmias, but this feature is not widely known or used among their millions of consumers. The aims of this paper were as follows: (1) to analyze the currently available sports heart rate monitors and assess their advantages and disadvantage in terms of heart rate and rhythm monitoring in endurance athletes; (2) to discuss what types of currently available commercial heart rate monitors are most convenient/adjustable to the needs of different consumers (including occasionally physically active adults and cardiac patients), bearing in mind the potential health risks, especially heart rhythm disturbances connected with endurance training; (3) to suggest a set of “optimal” design features for next-generation smart wearable devices based on the consensus opinion of an expert panel of athletes, coaches, and sports medicine doctors. Ninety-two experts aged 20 years and over, involved in endurance sports on a daily basis, were invited to participate in consensus-building discussions, including 56 long-distance runners, 18 cyclists, nine coaches, and nine physicians (sports medicine specialists, cardiologists, and family medicine doctors). The overall consensus endorsed by these experts indicates that the “optimal” sports heart rate monitor should be a one-piece device of the smartwatch type (with two or more electrodes), with integrated smartphone features, and able to collect and continually transmit data without exhibiting artifacts. It should continuously record at least a single-lead electrocardiography, send an alert after an unexpected fall, be of reasonable weight, come at an affordable price, and be user friendly.

## Plain Language Summary

Regular endurance training is among the key factors positively influencing human health. However, there are also many reports describing sudden cardiac fatalities and other serious health problems related to strenuous exercise. Millions of professional and leisure-time athletes worldwide use various digital heart rate monitors to keep track of their training volume, intensity, energy expenditure, running or cycling speed. Nevertheless, currently available heart rate monitors are not tailored to all the specific needs of their users. Moreover, they are not constructed to keep better track of important health functions and thereby improve the safety of endurance training. This paper presents a set of consensus statements developed by a panel of expert heart rate monitor users (endurance athletes, their physicians, and coaches) from three countries. In the panel’s view, the “optimal” heart rate monitor should improve users’ safety during exercise by providing more reliable medical data informative of potential health risks. A specific set of features of the proposed “optimal” digital heart rate monitor was identified and accepted by the panel of experts. Based on the consensus statements and the available literature, the authors propose next-generation portable devices for use by professional and ambitious leisure-time endurance athletes or even by cardiac patients involved in exercise training (a design called the “Gajda Watch” after the surname of two of the panel organizers/paper authors). One of the biggest challenges is dealing with artifacts and avoiding false information. This consensus document may also be helpful for manufacturers of heart rate monitors seeking new solutions for the sports and medical community.

## Key Points

**Table:** 

We report a consensus developed by a panel of expert users of heart rate monitors, including endurance athletes, their physicians, and coaches.
This consensus posits a set of “optimal” design features for a next-generation heart rate monitors for use by professional and leisure-time endurance athletes, as well as by cardiac disease patients, to improve users’ safety during exercise.
Two of the most important features of this single device indicated by the expert panel are artifact resistance and continuous transmission of electrocardiography recordings from at least one lead.
Appropriate design and user friendliness are also viewed by the panel as very important.

## Introduction

A heart rate monitor (HRM) is a personal monitoring device that allows the user to measure or display their heart rate (HR) in real time, and/or to record their HR data for later study. Such devices are largely used for gathering HR data during various types of physical exercise [1]. Observing an individual’s radial-artery pulse has long been used by physicians as a factor in diagnosis, since well before the advent even of classic watches, with no connection to competitive sports [2]. The pulse watch was first made commercially available by an English physician in 1701 [3]; partially reliable HR monitoring during training became possible over 200 years ago, with the spread of sweep hand watches. Modern HRMs first appeared in the 1970s [4].

Modern HRMs most commonly record heart signals by means of one of two methods, electrical or optical. The electrical approach is utilized by electrocardiography (ECG) sensors, which gauge the bio-potential generated by electrical signals in the body, whereas the optical approach is implemented by photoplethysmography (PPG) sensors, which use light-based technology to gauge the blood volume being moved by the pumping heart [5]. Electrical HRMs consist of two elements: a monitor/transmitter, predominantly worn as a chest strap, and a receiver. When the monitor detects a heartbeat, it transmits a radio signal, which is then used by the receiver to determine and display the current HR. This signal sent by the chest strap can be a simple radio pulse or be uniquely coded. More recent optical HR-measuring devices, in turn, use a light-emitting diode to emit light through the skin and measure how it scatters off blood vessels [6]. Some such devices are able to monitor HR while simultaneously measuring oxygen saturation and other parameters. They may also incorporate other sensors, such as accelerometers, gyroscopes, and global positioning systems (GPS), in order to detect and monitor speed, location, and distance [7].

In recent years, smartwatches have increasingly come to include HRM functionality, vastly boosting their popularity. Such smartwatches, as well as smart bands and cell phones, often use optical PPG sensors [8]. Currently, many vendors sell products with fitness and HR measurement technologies, predominantly using their own proprietary HR algorithms. Moreover, devices with ECG recording capabilities are becoming increasingly popular among physically active people, both with on-demand (e.g., Apple Watch) and continuous recording options (e.g., Frontier X2) [9]. Heart rate variability (HRV) is a function available in devices using both the ECG and PPG techniques. For instance, HRV data collection functionality were introduced to Apple Watch devices in 2018 [10].

At present, there are various types and a large number of portable medical devices for HR monitoring used by athletes, leisure-time exercisers, and cardiac patients. However, only a few are certified for use as medical devices [11]. Almost all currently used HRMs are capable of “catching” arrhythmias [12]. The main function of an HRM device is to monitor sports training, distance covered during practice, and pace. A high degree of accuracy and reliability is expected by endurance athletes. This requires not only GPS functionality but convenient control of these parameters as well, which should not interfere with or interrupt training. Heart rate rhythm monitoring is largely seen as an extra functionality by (presumably) healthy semiprofessional and professional endurance athletes, but it is perhaps considered the most important from the point of view of athletic club doctors, and also from the standpoint of the present article. The objectives of this paper, therefore, were as follows: (1) to analyze the currently available sports HRMs and assess their advantages and disadvantages in terms of HR and rhythm control in endurance athletes; (2) to provide expert indications, in the form of an overall consensus statement, of the commonly available functions that a sports HRM should have (in order to be as comfortable as possible, while also supplying thorough information for the purposes of identifying possible arrhythmias in endurance athletes during exercise and at rest); and (3) to educate the sports community about the cardiovascular risks in athletes and the possibility of risk self-detection using commonly available sports HRMs.

## Methods

In working to develop an overall consensus statement on the above issues, we recognized that even the most respected consensus statements have certain limitations [13] and none of the methods for adopting a consensus statement is universal (such as Delphi, non-RAND modified Delphi, the RAND/UCLA appropriateness method, the novel group awareness and consensus method) [14]. The method we chose for working with this expert panel is the nominal group technique (also known as the expert panel technique) [15].

We first identified an expert panel to be consulted on the functions of an “optimal” HRM for endurance athletes and to formulate a set of expert statements on sports HRMs in terms of assessing cardiac arrhythmias. Ninety-two individuals involved in endurance sports on a day-to-day basis were selected as experts for the present study, including 74 athletes (56 long-distance runners and 18 cyclists), nine coaches, and nine physicians (i.e., sports doctors, cardiologists, and family doctors), all of them aged 20 years and over. These 92 individuals were selected from among an original group of 138 candidates, all of them athletes, coaches, or physicians collaborating with the Center for Sports Cardiology at the Gajda-Med Medical Center in Pułtusk, Poland and all having over 6 years of experience in using electrical and/or optical HRMs. The inclusion criteria were as follows: (1) becoming familiarized with a selected body of required literature on cardiac arrhythmias and the technical capabilities of the different types of HRMs used by athletes, patients undergoing cardiac rehabilitation, and individuals leading a healthy lifestyle; (2) not having a sponsorship arrangement of any type with any sports HRM brand; (3) participation in a lecture session on different types of HRMs, symptoms of arrhythmias, and their risks for athletes; and (4) participation in the consensus-building panel discussion. Forty-six of the 138 candidates did not meet all four inclusion criteria (i.e., due to their inability to read the body of literature, their use of certain sports HRM brands because of sponsorship, or their declared inability to participate in the lecture or panel discussion), and thus were excluded from the panel.

The number of athletes on the panel was greater than for the other types of participants (representing 74 out of 92 total participants), meaning that there was an imbalance in favor of athletes’ preferences; however, they are potential future HRM users. Doctors were included in order to justify the expected functions of assessing HR and rhythm in the context of the safety of the athletes and the ability to implement the training plans set by the coaches effectively. Therefore, we decided to evaluate HRM features not by group (athletes/physicians/coaches), but according to the overall number of votes given by all panel participants in favor of a particular feature.

To select the body of literature for consensus participants, an electronic search was performed on the PubMed/MEDLINE and SPORTDiscus databases via EBSCO to find all English-language articles published between 1 January 2013 and 31 January 2023. These articles were extracted and reviewed by the consensus committee organizers (RG, JG, and WD), and prioritized in this order: systematic reviews, clinical practice guidelines, and original research. The keyword searches used included combinations of the following terms: [“Heart Rate Monitors”] AND [“arrhythmia” OR “palpitations”] AND [“artifact OR noise”] AND [“athletes”]. This led 48 studies to be identified in the bibliographic databases. Of these, articles that mentioned “Heart Rate Monitors,” “Arrhythmia,” and “Artifacts” in a sports-related context were included for review. As a result, a total of 18 articles were selected for the review; all others were excluded.

The nominal group technique was adopted as the consensus-statement selection method [15], as follows: (a) participants discussed scientific evidence dealing with particular issues; (b) participants formulated general statements expressing opinions or recommendation concerning HRMs, which were then voted on, and (c) proposals for individual features of the “optimal” sports HRM were selected and voted on. “Consensus” was considered to mean the view shared by the largest number of interviewees on any given aspect of the HRM device, including features, functions, appearance, and placement. As such, any statement receiving the largest number of votes of support among the expert panel was adopted as a consensus statement (Table 1). We used the AGREE II tool, which is reliable and widely used in the assessment of methodological rigor and transparency, to conduct an internal assessment of the soundness of the activities [16]. Voting was anonymous, and each individual participant’s vote was weighted equally.

**Table 1:** Categories of consensus statement

Consensus statement	Definition	Symbol
Approved by panel	Feature, description, opinion receiving the most votes of support among the panel of experts	✔
Disapproved by panel	Feature, description, opinion receiving the least votes of support among the panel of experts	**X**

The expert panel had no outside funders or funding. No conflicts of interest, commercial or intellectual, which may have influenced the decision-making process, were declared. The independence of the experts was ensured at all stages of developing the consensus statement, including during the anonymous voting. The impact of the consensus on clinical practice and athlete health could be positive, but will at worst be indifferent in both instances. We expect that the consensus decision reported herein may have an impact on future research on sports HRMs.

### Equity, Diversity, and Inclusion Statement

We invited our panel of endurance athletes, doctors, and coaches to engage in the consensus-building discussion without limiting the participation of people of different races, ethnicity, disability, nationality, socioeconomic stratum, sex, gender identity, and sexual orientation. Each participant had equal access to the literature and participation in a lecture session, and during the discussion and voting, each participant had an equal voice. The medical specialists included some physicians without academic titles and some with doctoral degrees, post-doctoral degrees, and the academic title of professor or full professor. The endurance athletes, in turn, included both leisure-time athletes, athletes from national teams, as well as leading athletes in international competitions. The invited participants were from three countries (Poland, Switzerland, and Ukraine).

## Results and Discussion

The full set of consensus statements approved or disapproved by our expert panel is listed in Table 2.

**Table 2:** All expert consensus statements approved or disapproved by the expert panel during discussion on sports HRMs

No	Feature, description, opinion	Panel approves/disapproves
1	The term “*digital devices for training control with HR monitoring”* is currently fully appropriate to refer to all devices with HR monitoring function for athletes and physically active people	✔ Panel approves
2	Optical HRMs are definitely less accurate when used during vigorous activity or when used underwater	✔ Panel approves
3	Sports HRMs should be “tailor-made” for each specific sport	✔ Panel approves
4	Arrhythmia provoked during extremely intense efforts (sports competition) still poses a diagnostic challenge for doctors and athletes	✔ Panel approves
5	Artifacts are the biggest problem that occurs when using HRMs and are sometimes the cause of incorrect decisions regarding a given athlete’s ability to continue practicing sports	✔ Panel approves
6	HRMs used by athletes should be excellent at monitoring the heart, even if that means they interfere with training and may adversely affect athletic performance	**X** Panel disapproves
7	Specifications for an “optimal” sophisticated sports heart monitor for endurance athletes, the “Gajda Watch”: One-piece device Waterproof Wristwatch with the features of a smartphone Hands-free function (to make a phone call) Camera to record the surroundings Artifact resistant Continuous transmission of ECG recordings of at least one lead On-demand alert function in case of symptoms Rechargeable once every 7 days Automatically sends an alarm signal in the event of a fall Ability to locate the user’s position Weighing no more than 50 g Round container shape Large high-contrast screen with large digits for reading data Data readable in all conditions of external lighting Signal in the event of unexpected increase in HR Algorithm to detect slow or fast paroxysmal atrial fibrillation Heart rate variability function Appropriate design which encourages frequent use No protruding parts (no scratching or snagging) Affordability Minimize inconvenience to the user Maintain quality (especially HR and ECG)	✔ Panel approves

*ECG* electrocardiography, *HR* heart rate, *HRMs* heart rate monitors

In the following sections, we summarize the expert panel discussion regarding the currently available sports HRMs, assessing their advantages and disadvantages in terms of HR and rhythm monitoring in endurance athletes. This discussion sets the context for the individual consensus statements proposed to the panel for approval/disapproval. Each of these consensus statements is discussed in turn.

### The Nomenclature for Devices that Monitor HR During Sports Activity

The first point of our panel discussion concerned the issue of nomenclature. Athletes have long used HR monitoring digital devices for training [17]. These devices are widely known as “heart rate monitors” or HRMs [18]. Originally, these mostly included sports watches equipped with GPS, which measured HR during a workout through straps with built-in electrodes placed on the chest, which transmitted the heart’s electric field data to a receiver inside the watch [19, 20]. Today—it was suggested in the panel discussion—it would be appropriate to modify the established colloquial name of these devices, as a multitude of models are on the constantly expanding market, with various designs and methods for recording both HR and training data. As for the mechanism used to record HR during training, two main types of devices have been dominant for years: PPG and ECG based [21]. Although it seems that these devices are still mainly associated with being a sports watch on the wrist, the location and shape of devices that measure physical activity and HR are practically unlimited, especially in amateur sports and among people leading healthy lifestyles. In particular, the devices for which the operating technique is based on the PPG mechanism can be housed in a variety of wearable gadgets, including earphones, rings, forehead bands, armbands, forearm bands, and leg bands [22]. Handheld devices, such as smartphones with face sensors and finger apps, are also becoming increasingly popular [23]. For some years, HRM electrodes have also been incorporated inside sports clothing, including bras and shirts [24].

It was suggested in the panel discussion that, given their existing variety, the name “*digital devices for training control with HR monitoring*” is fully appropriate for use as a cover term for all these devices. However, the ingrained name “*sports heart rate monitors*” (sports HRMs) also remains appropriate when referring to multifunctional digital training monitoring devices used by athletes.
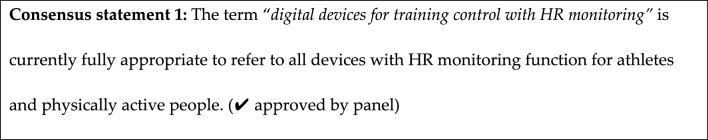


### The Reliability of Optical HRMs

The next point of discussion concerned the reliability of optical HRMs. Wrist-worn HRMs, although newer, have managed to attain almost identical levels of accuracy as their chest-belt counterparts; independent tests have reported up to 95% accuracy (although an error of over 30% may admittedly sometimes persist for several minutes [25]). Nevertheless, optical devices are definitely less accurate when used during vigorous activity [26] or when used underwater*.*

Previous research has identified several factors that affect PPG recordings, including the measurement site (i.e., probe attachment site), the contact force, mechanical movement artifacts, subject posture and breathing, as well as ambient temperature [27]. Photoplethysmography sensors can operate more effectively if they are placed at specific easily accessible anatomical positions, such as the earlobe and fingertip, where the desired PPG signals are collected with higher quality. Photoplethysmography sensors are designed in two different distinct forms: transmission mode and reflectance mode. For transmission mode, the fingertip and earlobe are commonly used. The measurement body placements for the reflectance mode sensors are the wrists, forearm, ankle, forehead, and torso [22]. However, one of the major difficulties in using PPG-based monitoring techniques is their inaccuracy in tracking the PPG signals during routine daily activities and light physical exercise. This limitation is due to the fact that the PPG signals are very susceptible to motion artifacts caused by hand movements [28]. The method by which the sensor is attached to the skin is also important (accuracy of location, compression strength). For example, an earring PPG sensor with magnetic attachment to the earlobe has been developed that allowed good contact for monitoring during physical activity [29, 30].
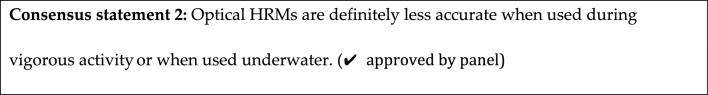


### Survey of HRMs Currently on the Market that are Potentially Useful for Diagnosing Arrhythmias in Athletes

The underlying mechanism by which sports HRMs operate is similar to that of other HRMs, used for example by patients with heart disease. These devices are also based on PPG and ECG technologies.

Photoplethysmography technology, the technique of shining light through the skin and measuring the amount that is scattered by the blood flow, is used in optical HRMs. In practice, these devices can only determine the HR without having the ability to record the ECG curve. Numerous algorithms created by individual companies producing optical HRMs allow for the diagnosis of atrial fibrillation (AF), with a high probability, based on HRV analysis; however, it is always necessary to confirm such a diagnosis with a standard ECG [22]. An HR recorder based on PPG can be placed almost anywhere on the body, keeping in mind the limitations of the device itself (which we discuss in Sect. 3.13 below). This has resulted in a plethora of different HR monitoring devices placed in watches, rings, wristbands, caps, other garments, earphones, and many other types. Data from these sensors are then sent to devices that collect and display them (e.g., smartwatches, smartphones, and PCs) [22].

For devices based on ECG technology, in contrast, data are obtained from electrodes that exhibit a difference in potential. Recording devices read the main electric field associated with the contraction of the ventricles (in most devices for athletes) and ultimately interpret this phenomenon of the beat-to-beat periods in the form of HR. If they have the function of measuring HRV (the variation in the beat-to-beat periods or variation in R-R intervals of the ECG curve), they become a helpful diagnostic tool in conduction disorders (measurement of pathologically long R-R intervals) [31]. In high-tech sports HRMs, a single- or multiple-lead ECG curve is recorded [32]. However, the electrode positions in standard ECGs are different from those used by wearable sensors, which moreover provide a reduced number of ECG leads that do not necessarily match a subset of the 12 standard ECG leads. This aspect influences the interpretation of ECG features acquired through wearable sensors [33]. When this electrical phenomenon is used by HRMs (main electric field registration) without ECG curve recording, the HR, which represents the peripheral effect of the heart pulse resulting from the frequency of contraction of the heart chambers, can be accurately determined without being able to assess the heart rhythm (sinus rhythm, AF, ventricular tachycardia [VT], or other) [34].

Countless ECG-based HRM devices have been developed. In these devices, the data are obtained from two electrodes that exhibit a voltage difference and allow the recording of HR and/or single-lead ECGs (e.g., Frontier X2). With three electrodes, a three- to six-limb ECG (KardiaMobile 6L) can be obtained [35]. These electrodes can be in a chest strap (Garmin Forerunner 910XT), in a handheld device (KardiaMobile 6L), or at any point on the body where a potential difference can be recorded (Life Signal Biosensor Patch) [36]. The potential for devices that do not yet exist on the market is now limited by the lack of suitable materials (breathable, flexible and stretchable materials, such as superflexible wood) as well as the limitations imposed by the laws of physics and engineering. Wearable devices are designed to be worn on different body locations for noninvasive sensing of an individual’s parameters without interrupting or restricting the user’s movements. Market research forecasts significant growth in the sport and fitness industry and heavy future investment in terms of industrial research, with the aim of improving sensors in terms of flexibility, motion, and the use of smart textiles [37, 38].

Some companies, e.g., Apple, have combined both techniques (PPG and ECG) in one device. When a user touches the Digital Crown of the Apple Watch Series 4, this completes the circuit, allowing the device to measure electrical signals across the heart (single-lead ECG, lead I: left hand-right hand). This function occurs on demand [39]. The ECG function of the Apple Watch, when worn on the wrist, is designed to monitor the electrical activity of the heart, but only in the direction of lead I, while ignoring the superoinferior axis captured by standard leads (II and III) and the horizontal plane captured by precordial leads (V1–V6) [40]. Independently, the same Apple Watch has a PPG sensor with an HRV function that continuously determines HR when the device is turned on (24/7). It has an irregular rhythm notification feature, which operates in the background and occasionally checks the user’s heart rhythm for signs of irregularity. If an irregular rhythm is detected on five such rhythm checks over a minimum of 65 min, an alarm sounds, and a notification appears [41]. Table 3 presents various portable devices based on ECG and/or PPG techniques that are, or could be, of practical use for amateur and professional athletes and/or people leading an active and healthy lifestyle.

**Table 3:** Heart rate monitors that could potentially be used during training or activity by amateur or professional athletes, or by people leading an active and healthy lifestyle

Device	Type	Area of application	Mode of detection	Cardiac sensor	ECG viewing/remarks	Recommended as usable and trustworthy for: P, A, HL, N
Garmin Forerunner 910XT	Wristwatch receiver	Chest strap with ECG device 2 E and monitor/transmitter	E-T HR	Only HR	NO	P, A, HL
Polar V800	Wristwatch receiver	Chest strap with 2 E and a monitor/transmitter	E-T HR (RR interval)	HR and HRV	NO	P, A, HL
Polar Vantage V	Wristwatch receiver	Chest strap with 2 E and monitor/transmitter + PPG in wristwatch	E-T and PPG-T HR only	Only HR	NO	P, A, HL
Garmin Fenix 5 wristwatch	Smartwatch	Wrist (option: + chest strap, HRM)	PPG-T HR	Only HR	NO	A, HL
Jabra Elite Sport earbuds	Smartphone	Ears	PPG	HR only	NO	A, HL
Motiv ring	Smartphone	Ring on finger	PPG	NO	NO	HL
Preventicus Heartbeats	Smartphone mApp camera	Fingertip	PPG	HR only	NO	N
Cardiio Rhythm	Smartphone mApp	Fingertip or video facial detection	PPG	HR only	NO	N
MOOV HR	Smartphone mApp with headphone in ear (active training)	Strap with PPG sensor on forehead sweatband (for most activities) or swim cap	PPG	HR only	NO	A, HL (works underwater)
Apple Watch	Smartwatch	Wrist-finger	ECG and PPG-T HR	2 E, 1 L ECG	On smartwatch and smartphone	HL
KardiaMobile 6L	Handheld	Fingertips ± leg or chest	ECG	2 or 3 E; 1 L or 6 L ECG	On smartphone, tablet, or PC	N
Wellue AI ECG Monitor	ECG recorder	Chest strap or ECG electrodes for connecting with the ECG recorder	ECG	1-L ECG	On tablet or PC	N
Frontier X2	Strap with ECG device	Chest strap with ECG device	ECG	1 L ECG	On smartwatch smartphone, tablet, or PC	P, A, HL (Ideal for extreme intensity sports, recommended in water)^a,b^
QARDIO MD	Smartphone mApp	Chest strap with 4 electrodes	ECG	4 E, 3 L	On PC	Currently clinical use only
Life Signal Biosensor Patch	Patch	Chest, self-adhesive	ECG	4 E; 2 L ECG	ECG on PC in-device, 5-day continuous measurement. Wireless near real-time telemetry and cloud	Medical device, single use only, CE and FDA validation Recommended for athletes with suspected arrhythmia during extreme exercises (e.g., competitions)^b^
Nuubo System-Shirt-Based	Recorder connected to shirt	Strap vest (shirt) on chest with electrodes	ECG	4 E 2 L ECG	Up to 30 days continuous recording, ECG on PC	P, A, HL

*A* amateur athletes, *CE* Conformité Européenne, *E* electrode(s), *ECG* electrocardiogram, *E-T* electrical techniques, *FDA* US Food and Drug Administration, *HL* healthy lifestyle, *HR* heart rate, *L* lead(s), *mApp* mobile application, *N* not applicable, *P* professional athletes, *PPG* photoplethysmography, *PPG-T* PPG techniques

^a^No validation or regulatory clearance

^b^The present authors’ recommendations

**Fig. 1:**
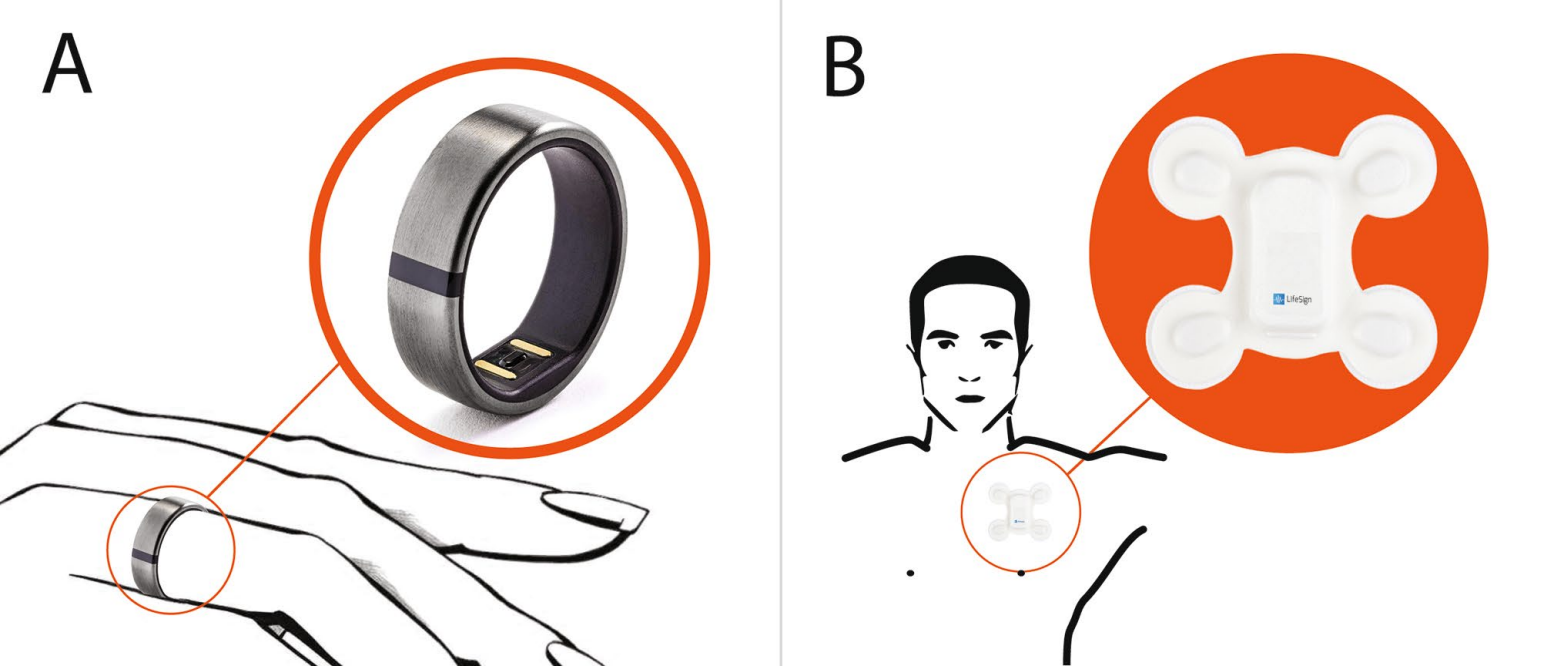
(**A**) Motiv ring and (**B**) LifeSignals Biosensor

Just as not all HRMs certified as medical devices are suitable for use by athletes, not all devices for athletes should be recommended for an accurate assessment of HR and rhythm. Of the hundreds of devices recommended by manufacturers as HRMs, only a small fraction provide reliable readings and are acceptably convenient for daily use during training [42]. On the contrary, better accuracy in a device does not always go hand-in-hand with convenience of use. In view of their convenience and comfort, wrist-based devices have largely replaced chest straps employing electrodes for measuring cardiac electrical activity. Although convenient, the Motiv ring (PPG technology) should not be recommended as a professional tool for monitoring HR. All the PPG-based devices have been shown to display poor HR agreement with one another during variable intensity running (Fig. 1A) [43]. In some HRMs, like the LifeSignals Biosensor, athletes can have their data transmitted wirelessly from the biosensor to a secure cloud-based platform with high reliability. Healthcare professionals in the athlete’s care continuum can then remotely access such data and rapidly make treatment decisions independently of the athlete’s actual location. However, it is a device for single use and not suitable for everyday training, although it can play a very important role when an athlete’s symptoms occur only with intense and long-term loads (e.g., when participating in semi-marathon, marathon, and ultramarathon competitions) [Fig. 1B].

With both PPG-based and single-lead ECG devices, it can be challenging to diagnose regular tachyarrhythmias from the atria, given their lack of (PPG) or difficult P-wave detection (ECG). It can be difficult to distinguish between AF, typical atrial flutter, atrial tachycardia, and junctional tachycardia, but the distinction is important when considering an ablation strategy [44, 45].

### HRMs Recommended for Athletes Practicing Specific Sports

It would seem to be quite natural that sports HRMs should be “tailor-made” for each specific sport. Commercial websites trying to meet the needs of athletes of different sports recommend different device types, citing different functionality, features, price, customer reviews, and vetting results [46]. After considering the sports HRMs used by athletes of various sports, one commercial website selected the “best” sports HRM for specific activities and categories as follows [46]:

- overall: Polar H10 Heart Rate Sensor;
- wrist: Fitbit Luxe;
- armband: Scosche Rhythm24 Waterproof Armband HRM;
- for swimming: Polar Verity Sense Optical Heart Rate Sensor;
- for running: Garmin HRM-Pro HRM;
- for cycling: CooSpo H808S Chest Strap HRM;
- multisport: Suunto Smart Heart Rate Strap;
- best Smartwatch: Fitbit Versa 3;
- most comfortable: Wahoo TICKR X HRM (list cited directly from source [46])

Although they cannot be used as indicators of quality and reliability, as they often lack a scientific basis in the form of relevant comparative studies, such commercial proposals strongly indicate that the “best” sports HRM would be one “tailor-made” for each athlete depending on a number of factors, including most importantly, the particular sports discipline(s) they practice. For example, in the case of HRMs assessing HR in swimmers, determining the accuracy of the device introduces additional challenges (e.g., the problem of setting up a Holter ECG to verify the accuracy of the indications) [47].
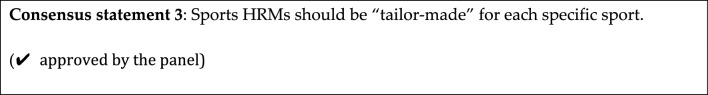


### Types of Arrhythmias in “Healthy” Athletes Detected on Sports HRMs

Athletes are not immune to any of the types of arrhythmias found in untrained healthy people or cardiac patients. Single ventricular or supraventricular beats are undetectable by most sports HRMs and are mostly non-threatening for athletes [48, 49]. It is more important to recognize the rapid rhythms that often occur at high physical loads [50]. Fast supraventricular and VT or rapid ventricular rhythms during paroxysmal AF in an athlete with pre-excitation syndrome can be dangerous [51]. Rapid supraventricular rhythms during training, such as recurrent atrioventricular nodal re-entrant tachycardia, are most often those that force the athlete to stop an exercise and are rarely the cause of syncope [52]. Paroxysms of AF or atrial flutter occur in athletes, both at rest and during exercise, but they have different mechanisms [53]. When they occur during exercise, it is impossible for the athlete to continue the effort, or they are at least forced to significantly reduce exercise intensity [54]. Individuals with AF and a rapid ventricular rate, or the need for long-term anticoagulation therapy, should be excluded from competitive sports [55]. An AF attack during the pre-excitation syndrome (Wolff–Parkinson–White syndrome) may be equal to hemodynamic cardiac arrest and, rarely (< 0.6% incidence), lead to sudden cardiac death [56]. A deadly danger is a VT, which can result not only in unconsciousness because of the heart’s hemodynamic dysfunction but also in an athlete’s sudden cardiac death [57]. A feature of all sports HRMs is that they can detect arrhythmia during training, which is the most important aspect. It is advisable for their morphology (supraventricular or ventricular) to be determined upon their occurrence. In practice, the use of digital devices for VT detection has lagged far behind their use for detecting AF, mostly because VT discriminators still require improvement. A sudden increase in pulse rate detected by a digital device is suggestive of possible paroxysmal tachycardias; however, most electrical and PPG HRMs are not able to discern the origins of tachyarrhythmia. Moreover, digital devices using ECGs need to be activated through an active process, which might not be possible to perform in non-tolerated VT cases [58]. Electrocardiography patches, which provide continuous recording with good quality records, are a specific exception in this respect; however, these are not typical sports HRM medical devices [59]. Future developments in wearable technologies may be expected to help diagnose symptomatic VTs, thereby aiding in clinical decision making.

### Dangerous Pauses and Bradycardias During Sleep Detected by Sports HRMs

Bradycardia is one of the adaptive features of exercise that is strongly expressed in endurance sports athletes [60, 61]. The low resting HR often observed by professional athletes also during the day is an indicator for them and for their coaches of increasing fitness as a result of training (increased athletic performance) [62, 63]. Nocturnal bradycardia of up to 30 bpm is also common in athletes and is still considered physiological [64]. However, when an amateur soccer player, for instance, records nocturnal pauses by way of sinus arrest of up to 7.3 s, expanding the diagnosis and/or offering therapy should be considered. Such pauses can be observed when a sports HRM has HRV functions (R–R variability), e.g., the Polar V800 [67]. Advanced bradycardia (< 30 bpm) can be recognized by all types of sports HRMs, without determining the cause (sino-atrial block, atrio-ventricular block, or other). The bradycardia mechanism can be identified on a sports HRM when the device records the ECG curve (e.g., Frontier X2) without waking the athlete from their sleep [65]. The ECG on-demand function is of no use in this case because waking up usually interrupts bradyarrhythmias. In any case, the athlete must sleep with an HRM (with at least a PPG sensor) or with a monitor or transmitter, typically worn in the form of a chest strap. In both cases, the transmitted data are collected by a receiver, such as a smartwatch or a smartphone lying at the bedside [66]. Frequent deconditioning is the main remedy for symptom resolution. If not, other therapeutic options should be considered [67].

### Recognition of Heart Conditions by HRMs

Sports HRMs are designed for healthy individuals engaged in recreational or competitive sports. Given this fact, is there truly a need to discuss the use of these devices in heart disease diagnostics? If we could be completely sure that every athlete is healthy, HRMs could be limited simply to assessing HR for training purposes. However, this is not the case, and every so often there are reports from various sports arenas of sudden deaths of prominent, seemingly “healthy” athletes during training or competition [68]. As cardiac ischemia during exercise can precede arrhythmias, it would be helpful if it could be recognized by sports HRMs. Some HRMs can record ECG continuously, without interrupting training. With the QARDIO MD, essentially a typical strap HRM, information from the transmitter (strap) is transferred to the receiver (a mobile app for iPhone), then sent to the data cloud after a delay of ~ 3 min. The Monitoring Center, which assesses the continuously recorded ECG data and automatically recognizes any life-threatening heart rhythm disorders, provides ECG recordings of three limb leads (modified leads I, II, III) with automated arrhythmia detection, QRS morphology analysis, P-wave detection (for enhanced automated AF detection), and the possibility of manually assessing the PQ, QT, and ST segments. This enables full control of the ECG recording with simultaneous medical supervision; however, the software is currently only available for physicians and hospitals [69].

An ECG ST segment evaluation is commonly used during exercise tests. Exercise test devices are automated, and medical personnel have vast experience in interpreting such tests. It seems logical, therefore, that it would be good for sports HRMs with ECG recording function to allow ST segment assessment; nevertheless, it should be borne in mind that if there is any possibility of ECG recordings being included in commonly available sports HRMs, they will be single-lead ECGs, which will not allow for a clear diagnosis. Additional artifact processing might not meet the requirements when compared with professional exercise test equipment with 12-lead ECGs or 12-lead Holter ECGs. Attempting to assess the ST segment or QT interval from sports devices may actually give rise to more issues (assessment of main artifacts, staff involvement, unnecessary stress for the athlete and coach) than potential benefits, namely, establishing a diagnosis of exercise-induced cardiac ischemia. However, we should not say no to future proposals for solving this important element of risk assessment in potentially healthy professional and leisure-time athletes.

### Extreme Exertion-Induced Arrhythmias Recognized by HRMs

At our Center for Sports Cardiology, one competitive athlete recently reported the following type of incident during a marathon run: “*When I ran 25–30 km at a minimum pace of 3:20 min/km in a competition, I would experience a sudden ‘cut-off’ with an increase in HR from* ~ *170 up to* ~ *220 bpm*” *(…) No training has ever provoked such symptoms.”* This professional athlete experienced a sudden increase in HR during a marathon run observed on a strap HRM (SUUNTO 9 PEAK), from 173–175 bpm to 210–225 bpm, together with a sudden decrease in exercise tolerance and a need to stop. The tachycardia resolved after ~ 1 min, and the athlete was able to continue running. Neither the exercise test nor the multiple Holter ECGs showed any exercise-induced heart rhythm abnormalities. A final electrophysiology study performed at a reputable center also failed to stimulate tachycardia and establish a diagnosis. [70].

The athlete in question, despite receiving a clear instruction to abstain from partaking in competitions and being informed about the associated risks, still does not intend to give up participating in professional sports. Therefore, it seems that his participation in a competition is also the right moment to look for diagnoses. So far, the other diagnostic tests performed for this athlete (ECG, transthoracic echocardiography, magnetic resonance imaging, and others) were within the normal limits for an “athlete’s heart.” In the near future, this athlete will take part in a marathon run (without our permission) and has asked about the most suitable HRM device that would have a reading reliable enough to establish a diagnosis and allow him to run freely. It seems that a 12-lead Holter would be the gold standard, if a recording undisturbed by motion could be obtained. However, the athlete’s comfort would be disrupted, and this would have a fairly significant impact on the outcome of the run, which is less important from the point of view of the potential risks he is exposing himself to.

**Fig. 2:**
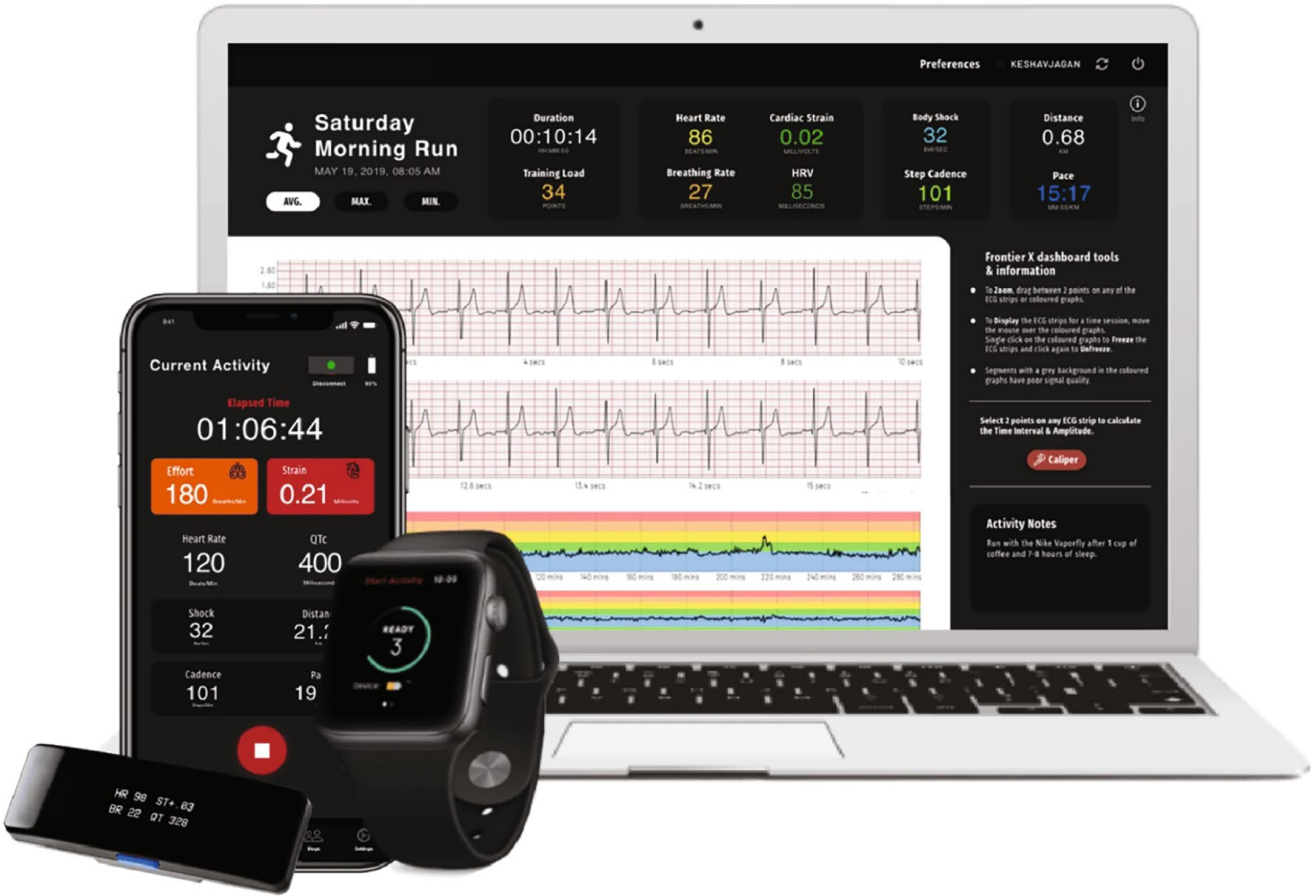
Frontier X2 heart rate monitor recorder, compatible with third-party devices (smartwatch, smartphone, and/or PC), connected via Bluetooth Low-Energy


We suggested that the athlete run with one of the following two devices: a typical medical device, the LifeSignals Biosensor Patch, or a sports HRM that records continuous ECG, the Frontier X2 (Fig. 2). The first suggestion, the LifeSignals Biosensor Patch (Fig. 1B), is a CE-certified device that is prescribed by healthcare professionals, providing continuous collection of patient ECG and physiological data in home or healthcare facility settings [71], transmitting data wirelessly, via a mobile phone, to a secure remote server. The device must be kept within 5 m (16 ft) of the biosensor and charged at least every 12 h to ensure continuous streaming of data [72]. Running with a smartphone will inevitably impact the athletic result, although this is not the most important matter in this context. The second suggestion was to run with the Frontier X2 HRM, a set that includes a recording device and a mobile app and web platform to monitor HR and rhythm, which is compatible with third-party devices (smartwatch, smartphone, and/or PC) and connected through Bluetooth Low-Energy. According to the manufacturer, the device is designed for extreme sports (including water sports), and the recorded data can be easily transferred to a mobile device or tablet immediately after a run. The device records a single-lead ECG on a chest strap recorder. Evaluation of the ECG on the “third device” in the sequence should be performed by a physician [9, 73]. We found no studies confirming the reliability of the recordings by the Frontier X2. Both devices appear to be relatively immune to artifacts from vibration during extreme exercise.

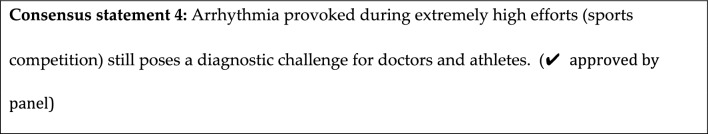


### Safety in Training, Above All?

Mobile apps that combine HR monitoring and analysis with the functions of fall detection, GPS positioning, video recording of the patients’ surroundings, and the ability to send alerts—triggered either by the detected symptoms or automatically, in the event of a detected fall—may be useful [74]. Loss of consciousness during training can have a variety of causes, and its consequences can be tragic if the training takes place on dangerous terrain (e.g., mountainous or marshy). Here, we focus on cardiac arrhythmia as a potential cause of losing consciousness. A function whereby the sports HRM automatically alerts an emergency number, friends, coaches, or teammates, with an option enabling the alert recipient to locate the transmitter, would seem reasonable and justifiable, especially when the alert can be immediately deactivated by the user (if it is a harmless trip and fall). Life-threatening injuries during training can occur not only due to cardiovascular or neurological causes (e.g., epileptic seizure) but also due to a collision with a vehicle or a blunt instrument (commotio cordis), or, not uncommonly, by an encounter with animals, such as a pack of dogs [75, 76].

### Artifacts: The Nightmare of Athletes and the Weakness of All Sports HRMs

As not all HRMs certified as medical devices (Conformité Européenne/US Food and Drug Administration) are suitable for use by athletes, not all HRMs used by athletes should be recommended for a reliable assessment of HR during particular heart rhythms. In fact, of the hundreds of HRM devices currently available, only a handful have a reliable HR reading that has been validated by studies and is acceptable for daily use during training [77]. The main concern on the part of athletes is the issue of unexpected changes in the values of HRM readings during training, which, when asymptomatic, mostly turn out to be artifacts [78]. Incorrect readings on HRMs may occur because of an immense number of factors that can affect data recording and/or subsequent transmission to the receiver. There are no HRMs completely immune to artifacts. Some are less sensitive (electrical), others more so (PPG) [9]. The biggest challenge for mobile device manufacturers is to work on reducing the likelihood of artifact occurrence and, thus, the inaccurate recording of HR and rhythm readings by HRMs.

There are many reasons for incorrect electric HRM readings during endurance exercise. Artifacts, interference, and noise during data transmission may occur at various stages of the HR monitoring process: at the level of the monitor or transmitter, which is worn mostly on a chest strap and contains electrodes, or at the level of the receiver, which is typically a device attached to the athlete’s forearm (e.g., a sports watch).

In the case of the chest strap, potential causes of interference include the following:

- poor adhesion between the HRM belt and the skin, especially during vigorous activity;
- a dead battery;
- an inadequately wet HRM belt, giving rise to greater interference at the beginning of training, before the athlete has begun to perspire;
- other factors, such as hair growing on the chest;
- bras worn by female athletes—this is an as-yet underappreciated problem causing interference in women; the lower edges of bras may sometimes deform the HRM straps running below them, and this disturbs conduction.

For the digital receiver, in turn, potential causes of interference include the following:

- the electromagnetic field produced by the strap may not be strong enough, or the digital receiver may be placed too far from the strap;
- thick clothing worn by the user (e.g., clothing designed for winter workouts, articles manufactured from materials such as nylon, or t-shirts made of synthetic materials, which may generate an electromagnetic field interfering with signals sent from the strap);
- exercising in the vicinity of devices that generate a strong electromagnetic field during training (e.g. electric traction devices);
- the proximity of another HRM worn by a different athlete, a mobile phone, a standard television, or any other electromagnetic field-generating device [78].

With PPG-based devices, interferences with HR measurement can also be abundant. The causes may include the following:

- cold body surface;
- muscle movements and tensing;
- high levels of vibration;
- water flowing under the optical HR sensor;
- sweating, darker skin, tattoos, or fatty tissue.

Table 4 summarizes the sources of the inaccuracy of PPG in continuous cardiovascular monitoring [79].

**Table 4:** Summarized noise sources and reasons for inaccuracy of photoplethysmography in heart monitoring

	Variable	Sources of noise
Individual variation	Skin tone	Absorption by melanin
	Obesity	Blood flow, skin thickness, capillary recruitment, transepidermal water loss, oxygen saturation
	Age	Skin thickness, vessel compliance, capillary recruitment
	Sex	Baseline cardiovascular differences, skin thickness, vessel size
Physiology	Respiratory rate	Low-frequency noise
	Venous pulsations	Reduced overall signal, low-frequency noise
	Local body temperature	Cold temperatures reducing PPG amplitude
	Body site	Signal amplitude and PPG waveform shape
External factors	Motion artifacts	High- and low-frequency noise
	Ambient light	Increased noise
	Applied pressure	Reduction in PPG amplitude and SNR

*PPG* photoplethysmography, *SNR* signal-to-noise ratio

Navalta et al. [43] reported that currently available PPG devices display poor HR agreement during variable intensity trail running. Until technological advances occur in PPG-based devices allowing for acceptable agreement, HR in outdoor environments should be obtained using an ECG-based chest strap that can be connected to a wristwatch or other comparable receiver. Gajda et al. [78] examined 142 regularly training endurance runners and cyclists, aged 18–51 years, with unexplained HR abnormalities indicated by various HRMs, in order to assess the utility of HRMs in diagnosing exertion-induced arrhythmias. Before reporting for these tests, most of the athletes were advised by their coaches or their doctors to refrain from competing until the diagnostics were completed. Two athletes were subjected to electrophysiologic tests, which did not reveal the cause of the possible arrhythmias observed on the HRM. Finally, significant rapid supraventricular tachycardia was confirmed in only one symptomatic athlete. In other athletes, the observed abnormalities indicated by HRMs were artifacts or no irregularities have been confirmed. Gajda et al. [78] concluded that the HRMs are not suitable tools for monitoring heart arrhythmias in asymptomatic athletes.
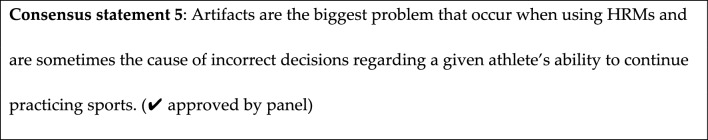


### Arrhythmias Observed on Sports HRMs in Symptomatic and Asymptomatic Athletes

**Fig. 3:**
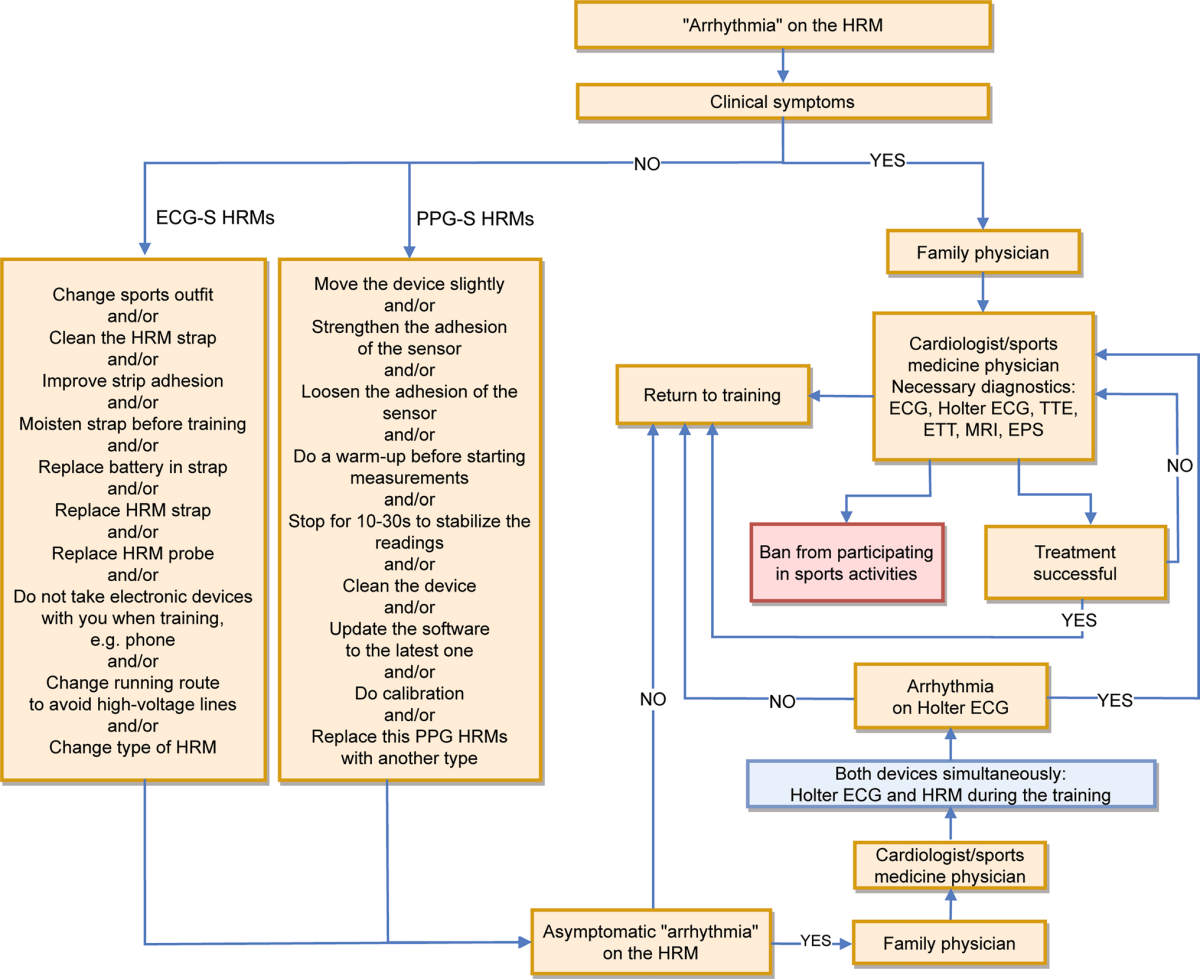
Procedure for managing suspected arrhythmias based on indications by different types of heart rate monitors (HRMs). *ECG* electrocardiography, *ECG-S* electrocardiography sensors, *EPS* intracardiac electrophysiology study, *ETT* exercise tolerance test, *MRI* magnetic resonance imaging, *PPG-S* photoplethysmography sensors, *s* seconds, *TTE* transthoracic echocardiography

A serious arrhythmia occurring during training, especially in the form of rapid tachycardia, cannot, in principle, be associated with an absence of symptoms. As a rule, when we are dealing with supraventricular tachycardias, such as atrioventricular nodal re-entrant tachycardia, the athlete is forced to stop training or significantly reduce the run intensity [52]. In the case of VT, the symptoms depend on its rate and the circumstances under which it occurred, e.g., during cardiac ischemia. They may involve loss of consciousness and, in extreme cases, cardiac arrest [80]. Atrial fibrillation occurs less frequently during exercise in healthy athletes, generally occurring at rest after meals or during sleep [81]. The heart’s single supplementary beats are virtually imperceptible during exercise. If an athlete observes a tachycardia attack on the HRM during exertion, e.g., in the form of a sudden HR increase from 160 to 220 bpm, and is able to continue exertion without accompanying physical symptoms (other than consequential psychological unease), it can most likely be concluded that the reading on the HRM is false [78]. Some prominent ultramarathoners, however, do not use HRMs when competing in sporting events, one reason that they cite being a lack of full confidence in their indications [82–84]. We propose a set of principles for managing abnormal indications on HRMs during exercise with or without accompanying clinical symptoms, as shown in Fig. 3

The methodology proposed by the present authors, which involves HR recording with a Holter ECG monitor concurrently with an HRM during training, is an excellent option for examining athletes in whom repeated asymptomatic episodes of unusually high or low HRs (arrhythmias) have been observed on HRMs during training. In most cases, the two recordings taken simultaneously are likely to reveal normal sinus rhythm, and this may reassure the athlete [78].

### What Should the Optimal HRM Created for Endurance Athletes Look Like?

Theoretically, competitive athletes are assumed, a priori, to be well screened and healthy and, therefore, not to be at risk of cardiac arrhythmias. If true, this assumption would certainly have cut short and rendered pointless our discussion on the need to work out optimal HRM characteristics. However, all the expert participants in our panel were well aware that more than one healthy athlete has experienced a cardiac arrest that they did not survive [68], and every athlete participating in the expert session had at least once been concerned about abnormal HRM readings during training, with or without accompanying symptoms. Despite the concern of doctors and coaches for the health of athletes, sports devices for monitoring the training of endurance athletes are primarily designed to monitor the covered distance and speed. The next parameter that needs to be monitored is HR, in terms of training load, i.e., its intensity for the body. Athlete safety, as expressed by the evaluation of the heart rhythm, is just another element for which sports HRMs are used [69]. As the analysis of assessable parameters regarding the workout would have gone far beyond the purpose of our panel discussion, it was agreed that in the jointly defined “optimal” HRM, the basic characteristics concerning the workout, such as speed and distance, would be calculated using GPS in the case of outdoor workouts. The device should calculate these and other parameters for indoor training based on the accelerometer, gyroscope, and pedometer [85]. Practice shows that HR and rhythm monitoring have been and continue to be additional features in athletes’ expectations of mobile devices for training control. Only such an approach could guarantee the use of the designed HRM by athletes during daily training. Such a device used by athletes, no matter how excellent it may be at “monitoring the athlete’s heart,” should not interfere with training and sports competition or have any adverse effects (even slight effects) on the athlete.
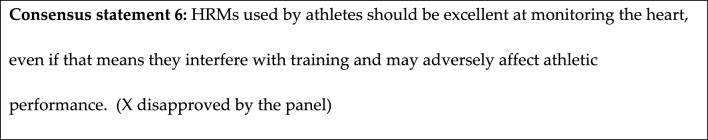


We discussed the proposed shape, placement, acceptable weight, number of elements that such an HRM (meaning: *digital devices for training control with heart rate monitoring*) should consist of, its physical features, how long the device should operate without needing to be recharged, ease of use, size of the displayed elements, and a number of other features. To the best of our knowledge, we analyzed individual features using pre-existing HRMs or proposed solutions not yet available on the market. By analyzing and discussing through consensus (checking whether there was a majority of votes in favor of each feature), we developed the specifications for an “optimal” HRM, as follows.

**Fig. 4:**
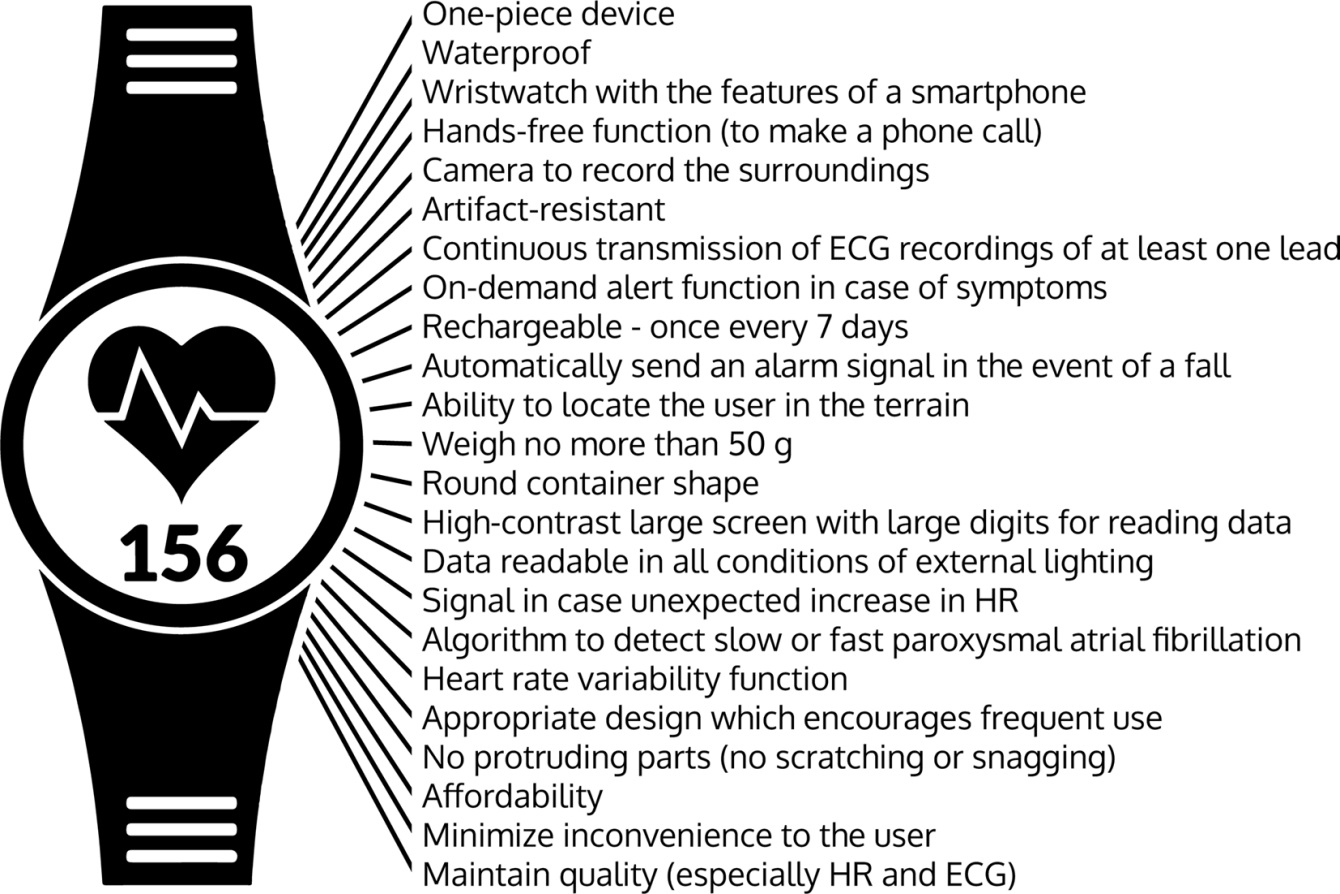
Attributes of a sophisticated sports heart monitor for endurance athletes, the “Gajda Watch”: an expert consensus. *AF* atrial fibrillation, *ECG* electrocardiography, *HR* heart rate

It should be a one-piece device—a waterproof wristwatch with the features of a smartphone (i.e., not requiring an additional device for data collection and transmission)—that has hands-free functionality (the ability to make a phone call) and a camera able to record the surroundings. This single device worn for training should be artifact resistant and provide continuous transmission of ECG recordings of at least one lead. It should have an on-demand alert function (to compare the subjective feelings of the athlete with the simultaneous objective ECG recordings by the club doctor by phone), not require recharging for at least 7 days, and automatically send an alarm signal in the event of a fall to designated persons (coach, teammate, family, and/or club doctor) with the possibility of allowing them to identify the athlete’s location. Furthermore, the athlete would need to be able to cancel such an alert. The device should weigh no more than 50 g, have a round container shape, and have a large high-contrast screen with large digits for reading data in all conditions of external lighting and by athletes of different ages. An unexpected increase in HR above an expected limit (e.g., above the maximum HR for the athlete), a sudden unexpected increase (e.g., by 10–20 bpm) over a short period (e.g., within 10 s), or a decrease below the predicted values (e.g., overnight pauses over 4 s or below 30 bpm) should trigger a signal to the athlete and their club doctor to verify the readings. The device should have an algorithm to detect slow or fast paroxysmal AF (from HRV function, verified by an ECG recording). The appropriate design should encourage frequent use both during and outside training sessions. As its constant use and wearability during sleep are preferable, it is important that it should not have protruding parts (no scratching or snagging). An indispensable attribute is affordability. We have proposed to call this design the “Gajda Watch”, not coincidentally echoing the name of two of the meeting organizers/authors of this article (Fig. 4 and Table 2**,** point 7).

We did not debate the mechanism of HR recording (PPG vs ECG-based techniques, electrode placement), recognizing that this should be left up to the discretion of the designers and not the users. It is important that the device should be as free of artifacts as possible, thus minimizing the inconvenience of controlling all parameters for the athlete while maintaining the quality of the recording (such that the diagnosis can be established in the event of cardiac arrhythmias), and be able to immediately notify the user and designated emergency services, including the club doctor.

### Perspectives, Doubts, and Limitations

Doubts have been raised because of the sheer excess of data collected and evaluated, which might lead to the unnecessary burdening of the doctors who will ultimately have to verify such data. Therefore, one might argue, does it even make sense to try to “make a patient out of a healthy athlete” and “make a medical device out of a sports gadget”? It is conceivable that every ECG from a workout will be sent in by an overzealous coach or athlete for evaluation by a doctor, who, in turn, lacking confidence in the readings recorded by the watch, will either ignore it or take action out of an uncertainty as to whether they are dealing with artifacts or actual heart rhythm disturbances. On this potential line of reasoning, it might be argued that a healthy athlete does not require permanent monitoring, while a sick athlete should be treated and should not risk a dangerous cardiac event during intense training. The main doubt that arose in the discussion of our group of experts tasked with “inventing” the optimal HRM was whether there is even a need to record parameters other than HR readings during training (e.g., attempting to assess cardiac arrhythmias). Theoretically, athletes undergo periodic examinations, and such subtle control of possible heart rhythm disturbances may seem unnecessary.

Counterbalancing these doubts are the occasional discussions about the need to place advanced and expensive devices, such as automatic cardioverter defibrillators, in various locations [86]. Although rarely used, they have nevertheless saved more than one life [87]. Portable devices, such as sports HRMs, are currently used by millions of people. Our team is currently starting a long-term observational study of patients with long QT syndrome type VII, employing modern HRMs for use in ultramarathons (with an extensive battery life) [88, 89]. In the near future, we intend to use HRMs to assess potential heart rhythm disturbances during endurance training under hypoxia [90]. It therefore seems sensible to add features, such as continuous ECG recording, to HRMs used by athletes in future devices for people with healthy lifestyles, or even for those with unhealthy lifestyles (perhaps even more so). It would also be worthwhile for HRMs to include life-saving functions for cardiac emergencies. This can be especially important during the resurgence of sports in the aftermath of COVID-19 (especially in professional athletes) [91, 92].

Another disadvantage of attempting to identify an “optimal” sports HRM in such an expert group is that the search was limited to athletes of endurance sports such as running and cycling. It is likely that athletes of other endurance disciplines, such as swimming and cross-country skiing, would report other characteristics they would expect from their sports HRMs with expanded cardiovascular functions, as would their coaches and club doctors. Technological development in the field of training monitoring devices with HR assessment functions is extremely rapid, and it cannot be ruled out that something like the “Gajda Watch” design proposed by us here as an “optimal” or “dream” sports watch will soon be a widely available type of device, only to become technologically obsolete soon thereafter.

This article has a number of limitations we are aware of. First, the methodology employed—using an expert panel as a method of developing a broad consensus statement—brings with it certain limitations (as does any method of creating a consensus). The scientific value of such a panel’s findings can be critically debated [13]. However, our counter response to this would be that we have made every effort to ensure that the method used here, the nominal group technique method, was applied in a way consistent with its guidelines. The various statements making up the consensus posited herein do not constitute treatment guidelines or negate existing opinions. Rather, they relate to sports HRMs and the expressed expectations of those who are experts on the subject.

Other limitations are related to the engineering realities of the proposed solutions posited for such an advanced device. The participants in the expert panel meeting were not trained as engineers designing medical apparatus or sensors used in medicine, and thus the ensuing discussion may not have taken into account various engineering problems that could potentially hamper or prevent the simultaneous functioning of the desirable features of the optimal device, as posited in the consensus (for instance, the placement and number of electrodes for recording ECG were not discussed).

## Conclusions

In this paper, we surveyed the currently available sports HRMs and assessed their advantages and disadvantages in terms of HR and rhythm monitoring in endurance athletes. We also discussed what types of currently available commercial HRMs are most convenient/adjustable to the needs of different consumers. Most importantly, however, our panel of endurance athletes, coaches, and physicians endorsed a set of statements that together make up an expert consensus regarding the design of an “optimal” sports HRM. The panel recognized optical HRMs as being definitely less accurate when used during vigorous activity or when used underwater, and that it is best for sports HRMs to be “tailor-made” for each particular sport. In terms of challenges, the panel agreed that arrhythmia provoked during extremely intense effort (sports competition) still poses a diagnostic challenge for physicians and athletes, and that artifacts are the biggest problem that occurs when using HRMs. The panel did not, however, endorse the notion that HRMs used by athletes should be excellent at monitoring the heart, even at the potential cost of interfering with training and performance.

As part of the consensus, the experts proposed a detailed set of design recommendations for an “optimal” sports HRM. Any HRM that an athlete will not actually use for training because of its specific limitations or inconveniences, e.g., weight or short battery life, will be of zero value. The voluntary use of an HRM (of the right design) guarantees that athletes will benefit from the expert consensus solutions proposed for HR and rhythm monitoring. As such, to ensure adoption and adherence, athletes need to be enlisted from the beginning of the development work, in order to co-design the devices while keeping the end user firmly in mind.
